# Excessive screen time and problem behaviours among school-age children in Fujian, China: a cross-sectional study

**DOI:** 10.1186/s12889-025-21795-4

**Published:** 2025-02-18

**Authors:** Yang Yu-ting, Chen Li-xiang, Yao Miao, Yang Yong-wei, Lin Ting

**Affiliations:** 1 The School of Nursing, Fujian Medical University, No. 1 Xuefu North Road, New University District, Fuzhou, Fujian 350122 China; 2 The Affiliated Hospital of Putian University, No. 181 Meiyuan East Road, Putian, Fujian China

**Keywords:** Child, Problem behaviour, Screen time

## Abstract

**Background:**

Screen time encompasses activities conducted on digital devices, including traditional devices such as televisions and computers, as well as modern devices like smartphones, tablets, and other digital screens. Excessive screen time among children has been linked to a heightened likelihood of engaging in high-risk problem behaviours. This study aimed to quantify the prevalence of excessive screen time and delve into its correlation with problem behaviours among school-aged children in Fujian, thereby gaining insight into the prevalence and trends within this region.

**Methods:**

From October to November 2022, we used cluster sampling and invited 891 school-age children from Fujian as participants. Parents recorded children’s screen time for a week, taking the average value of seven days, and the unit is minutes. The Child Behaviour Checklist was used to assess their problem behaviours. Correlation analysis, Propensity Score Matching, Single-factor analysis, and Multi-factor analysis were used to explore the influencing factors and correlation.

**Results:**

Screen exposure time of school-age children was 34.29 (17.14, 55.71) min/day. Then, 17.06% were exposed to excessive video. The total CBCL score of school-age children was 10.00 (3.00, 22.00), and 10.00% had problem behaviours. After matching the propensity score, the total score of problem behaviours in the daily over-exposure group was higher than that in the non-over-exposure group (*Z* = 5.466, *p* < 0.001). Generalized linear model analysis showed that after controlling confounding variables, daily video exposure time or daily excessive screening could affect problem behaviours (*p* < 0.05).

**Conclusions:**

The incidence of problem behaviours among school-age children is low in Fujina. The daily screen time, weekday screen time, and weekend screen time were positively associated with problem behaviours. We suggested family members shift to more serious and cautious attitudes toward children’s screen exposure and adopt appropriate digital-related parenting practices, such as accompanying children when they inevitably need to watch electronic devices, to better understand and manage their screen time.

**Supplementary Information:**

The online version contains supplementary material available at 10.1186/s12889-025-21795-4.

## Background

Up to 20% of children and adolescents worldwide experience problem behaviours [1]. Problem behaviours exhibited by school-aged children pose significant obstacles to their healthy growth and development, warranting careful attention. These behaviours, which encompass abnormal behaviours that impede social adaptation and undermine children’s normal psychological and physical health, can be broadly categorised into externalised and internalised problem behaviours. Externalised behaviours include aggressive and disciplinary violations, while internalised behaviours pertain to emotional and affective issues, such as anxiety and depression [2].

Research indicates varying rates of problem behaviours detection among children and adolescents globally. In Sri Lanka, the rate is 13.80% [3], while in China it spans from 6.5 to 17.6% [4–6]. A 2021 Chinese study [4] involving 71,929 youths aged 6–16 reported a 17.6% prevalence of such issues, and another from Zhengzhou in 2022 found 7.8% of primary school students displaying problem behaviours [7]. Over the past three decades, there’s been a rising trend in these behavioural and emotional problems [5–7]. These issues significantly impair children’s development, affecting learning [8], relationships [9], and potentially leading to personality and mental health disorders [10]. Unfortunately, they are often overlooked by caregivers, missing early intervention chances and negatively impacting growth [11]. Identifying and addressing the factors influencing these behaviours is crucial for improvement.

Using screen media devices is prevalent among 21st-century children [12], with a study [13] showing excessive use linked to emotional issues, problem behaviours, hyperactivity, and peer conflicts. A significant difference in rule-breaking behaviour scores was found between children with high vs. moderate screen time (*p* < 0.05) [14]. Moderate screen time correlates with fewer emotional symptoms and conduct problems [15]. Activities like watching TV or movies are associated with increased rule-breaking (5.9%), social issues (5%), aggression (4%), and cognitive difficulties (3.7%) [16]. Video game duration correlates with physical symptoms and aggression severity [16]. The Child Behaviour Checklist (CBCL) effectively measures the impact of screen time on behavioural issues; for example, it showed higher problem behaviour rates in preschoolers with excessive (36.4%) vs. moderate (30.8%) screen time [17]. Similarly, Teng’s analysis of 2131 children aged 4–6 years revealed that average daily screen time was a significant factor influencing children’s problem behaviours [18]. These findings underscore the importance of monitoring and managing children’s screen exposure to mitigate adverse effects.

Screen time encompasses activities conducted on digital devices, including traditional devices such as televisions and computers, as well as modern devices like smartphones, tablets, and other digital screens [19]. An analysis of screen time and problem behaviours among American children revealed that out of 101,350 participants, 13,156 (13.0%) were reported to have less than one hour per day of screen time, 16,606 (16.4%) had one hour per day, 28,997 (28.6%) had two hours per day, 19,533 (19.3%) had three hours per day, and 23,058 (22.8%) had four or more hours per day [20]. Wu’s 2016 survey of 43,771 students reported excessive screen time prevalence of 16.2% on school days and 41.5% on weekends, with respective medians of 0.9 and 1.8 h [21]. Indigenous Canadian children, particularly Inuit, often exceed the recommended limit of one hour daily [22]. The surge in internet use and video devices raises worries about links between excessive screen time and negative child behaviours [16, 23].

International research highlights screen time’s impact on children’s behaviour. A meta-analysis found modest correlations between screen time and problem behaviours [24], with Indigenous Canadian youth experiencing heightened socioemotional and behavioural challenges [22]. Younger Japanese children are more susceptible to mobile device influences than older ones, potentially exhibiting oppositional behaviours typically seen in teens [25]. US data indicates excessive screen time correlates with developmental and behavioural risks, especially in preschoolers and boys [20]. Similarly, a large Chinese study connected excessive screen time to both internalizing and externalizing behavioural issues among Shanghai children, underscoring the need for controlled screen exposure in students [26].

The global rise in problem behaviours among school-aged children varies by country and includes both externalized (aggression, disciplinary issues) and internalized (anxiety, depression) behaviours. Excessive screen time is associated with emotional problems, problem behaviours, hyperactivity, and peer conflicts. Previous research often relied on estimations or self-reports, which may not accurately reflect true screen time usage. Our study used caregiver observations to record actual screen time over a week, providing direct data on electronic device use from Monday to Sunday.

In short, the investigation into the association between screen time and problematic behaviors among school-age children remains underexplored. To advance this field, it is imperative to control for confounding variables more rigorously, thereby facilitating a deeper understanding of the relationship between these two factors. This will contribute to the evidence base for analyzing the correlation between screen time and problematic behaviors. Prior research has indicated a potential link between screen time and problematic behaviors in children; however, the body of research in China is inadequate. Specifically, (a) the onset of such studies was delayed, and the sample sizes were insufficiently large; (b) there remains considerable scope for exploring and valuing the impact of “screen time” on children; (c) the findings from studies on school-age children in China require updating and supplementation. We conducted a comprehensive analysis of the link between excessive screen time and problem behaviours, aiming to elucidate their impact and offer empirical evidence for addressing these issues.

## Materials and methods

### Study design and participants

#### Participants

This study employs a multi-stage stratified cluster sampling method for the investigation, which consists of four stages. Firstly, Fujian Province currently administers nine district-level cities: Fuzhou, Xiamen, Putian, Quanzhou, Zhangzhou, Longyan, Sanming, Nanping, and Ningde, as well as the Pingtan Comprehensive Experimental Zone. A simple random sampling method was utilized to select Putian City for the investigation. Secondly, based on the administrative divisions, one administrative region from Putian City, including its municipal districts, county-level cities, and counties, was selected using a simple random sampling method. Thirdly, within the selected administrative region, all primary schools were considered as units, and seven schools were randomly selected using the Random Number Generator website (https://www.randomizer.org/). Lastly, a cluster sample survey was conducted among the seven selected primary schools.

The public primary schools we selected are from the jurisdiction of the Fujian Meizhou Island National Tourism Resort Management Committee, supported by administrative support. The study design was reviewed and approved by the Ethics Review Committee of the Affiliated Hospital of Putian University (Approval No. 202223) on September 3, 2022. This study followed STROBE guidelines. Data collection commenced only after obtaining consent from the children and their parents. In the meantime, our study adhered to the Declaration of Helsinki. Furthermore, we ensured that each questionnaire was thoroughly reviewed, and those that did not meet the required standards were excluded.

#### Sample size

The dependent variable in this study was the total score for problem behaviours, which was classified as measurement data. This study employed simple random sampling to estimate the population mean, utilising the sample size calculated using the formula:

$$n_{1}\;=\;\left(u_{a/2}\;\times\;\sigma/\delta\right)^2$$

If *α* = 0.05, then *u*_α/2_=1.96. According to the pre-experimental results, the total score *M* (*P*_25_, *P*_75_) of problem behaviours in 66 school-age early children was 6.00 (0.00, 18.25) points, with a mean value of 13.95, and a standard deviation of 27.38. The allowable error was set at 10% of the total standard deviation, resulting in an *n*_*1*_ of 384 cases.

Subsequently, cluster sampling was adopted, and the sample size for the cluster sampling study was adjusted based on the design effect (deff), calculated as follows:

$$n_2\;=\;deff\;\times\;n_1$$

Deff typically ranges between one and three. In this study, if deff = 2, *n*_*2*_ was calculated to be 768, accounting for a 10% non-response rate and a minimum of 853 samples was required.

#### Inclusion and exclusion criteria

First and second grade students from seven public primary schools were invited to participate in this study. Following previous studies [27, 28], our team discussed and formulated the following inclusion and exclusion criteria. The inclusion criteria were children aged 6–9 years currently enrolled in school and whose caregivers could comprehend and express simple Chinese characters. Children with severe illnesses, including congenital heart disease, autism, intellectual disabilities, or other specific brain disorders, were excluded from the study. Informed consent to participate had obtained from the parents or legal guardians of any participant under the age of 16. Researchers contacted their parents, enquired about their willingness to participate, and obtained written informed consent. The children and their parents were assured that their privacy would be respected and that the information provided would be solely for research purposes. Questionnaires were stored securely in a locked file cabinet. Between October and November 2022, 936 parents were invited to complete the questionnaire. Upon completion, we checked the questionnaires for missing answers or obvious logical errors, and incomplete questionnaires were excluded from analysis. A total of 891 parents participated, yielding a response rate of 95.19% (891/936).

### Measurements

We evaluated the features, applicable population, convenience, availability, and/or reliability and validity, etc., of the questionnaires and finally selected them based on the purpose of our study.

#### Participants demographic characteristics

The self-designed questionnaire consisted of questions pertaining to age, sex, grade, nationality, singleton status, overweight or obese status, birth order, primary caregiver, parental educational level and occupation, and per capita monthly household income.

#### Smartphone Use Questionnaire (SUQ)

The SUQ, based on a questionnaire originally designed by Shen [29] and adapted by Liu [30], primarily consist of eight items that objectively record behaviour: assisted learning, socialising (connecting with others), browsing web pages, playing mobile games, watching movies and videos, listening to music, reading e-books, and taking pictures or shooting videos. Respondents were asked to indicate the frequency of these activities using a scale of 1 to 5, where 1 indicates “never”, 2= “occasionally”, 3= “sometimes”, 4= “often”, and 5= “always”. The average score also ranges from 1 to 5, with higher scores indicating more frequent use of smartphone. The contents of the SUQ have been provided as a Supplementary File. This questionnaire objectively records behaviour. After consulting with statisticians, we decided not to conduct reliability and validity tests.

#### Screen time questionnaire (STQ)

The STQ was designed based on the concept of Ge [31]. Caregivers observed children at home for a week and recorded the time spent watching television or using electronic devices, such as computers or mobile phones, from Monday to Sunday. The evaluation measures included daily screen time, weekday screen time, weekend screen time, and excessive screen time. Daily screen time was calculated as the total screen time divided by seven. The weekday screen time was equal to the total weekday time divided by five. The weekend screen time was determined by dividing the total weekend screen time by two. In this study, more than 1 h per day was considered as excessive screen time [32]. The content of the STQ has been provided as a supplementary file. This questionnaire objectively recorded behaviour. Following consultation with statisticians, we decided not to conduct reliability and validity tests.

#### The child behaviour checklist (CBCL)

The CBCL, originally developed by Achenbach in 1976 [27, 28], was introduced in China in 1988 by Xin et al. [33] for the assessment of problem behaviours among children aged 4–16 [34]. This study focuses on evaluating problem behaviours among children aged 6 to 9. Table 1 details the items associated with each dimension, along with the conventional upper limit [34] and the upper limit adopted in this study. The scale comprises 113 items and 120 questions. The parents rated the items on three-point response scales: 0 indicating that the item was not true for the child, 1 indicating that the item was somewhat or sometimes true for the child, and 2 indicating that it was very true or often true for the child. For male students, the dimensions included schizoid behaviour, depression, uncommunicativeness, obsessive-compulsive symptoms, somatic complaints, social withdrawal, hyperactivity, aggression, and delinquency. Female students were categorised into dimensions such as somatic complaints, schizoid-obsessive symptoms, depression, social withdrawal, sexual problems, cruelty, delinquency, aggression, and hyperactivity. The evaluation indices employed in this study were as follows: (1) total score, calculated as the sum of scores from all 120 questions, with a higher total score indicating a greater prevalence of problem behaviours among children; and (2) each dimension, determined by comparing scores in the nine dimensions for boys or girls against the upper limit adopted in this study (as outlined in Table 1). Where the scores exceeded this limit, problem behaviours were considered present in that dimension [34]. The CBCL has demonstrated good reliability in Chinese populations [35–38], with a retest reliability of 0.95 within a week, parental evaluation consistency of 0.97, and a *Cronbach’s α* coefficient of 0.872 [34]. The *Cronbach’s α* coefficient for the CBCL of this study was 0.963. The contents of the CBCL have been uploaded to a supplementary file.

**Table 1:** Dimensions, items, and upper limits for CBCL

Gender	Dimensions	Number of items	Attribution of items	Scores	Conventional upper limit	Upper limit of this study
Total sample	CBCL	113	All items	0 ~ 240	37 ~ 41	37
Boys	Depressed	17	12, 14, 18, 31, 32, 33, 34, 35, 45, 50, 52, 71, 88, 89, 91, 103, 112	0 ~ 34	9 ~ 10	9
	Somatic Complaints	9	49, 51, 54, 56a, 56b, 56c, 56f, 56 g, 77	0 ~ 18	6 ~ 7	6
	Hyperactive	11	1, 8, 10, 13, 17, 20, 41, 61, 62, 64, 79	0 ~ 22	10 ~ 11	10
	Aggressive	23	3, 7, 16, 19, 22, 23, 25, 27, 37, 43, 48, 57, 68, 74, 86, 87, 88, 90, 93, 94, 95, 97, 104	0 ~ 46	19 ~ 20	19
	Delinquent	12	20, 21, 23, 39, 43, 67, 72, 81、82, 90, 101, 106	0 ~ 24	7 ~ 8	7
	Social Withdrawal	8	25, 34, 38, 42, 48, 64, 102, 111	0 ~ 16	5 ~ 6	5
	Obsessive-compulsive	16	9, 13, 17, 46, 47, 50, 54, 66, 76, 80, 83, 84, 85, 92, 93, 100	0 ~ 32	8 ~ 9	8
	Uncommunicative	8	13, 65, 69, 71, 75, 80, 86, 103	0 ~ 16	5 ~ 6	5
	Schizoid	9	11, 29, 30, 40, 47, 50, 59, 70, 75	0 ~ 18	5 ~ 6	5
Girls	Depressed	18	11, 12, 30, 31, 32, 33, 34, 35, 38, 45, 50, 52, 71, 75, 88, 103, 111, 112	0 ~ 36	12 ~ 13	12
	Somatic Complaints	13	2, 47, 51, 54, 56a, 56b, 56c, 56d, 56e, 56f, 56 g, 77, 92	0 ~ 26	8 ~ 9	8
	Hyperactive	14	1, 8, 10, 13, 17, 23, 38, 41, 48, 61, 62, 64, 79, 80	0 ~ 28	10 ~ 11	10
	Aggressive	23	3, 7, 14, 16, 19, 21, 22, 23, 25, 27, 33, 37, 41, 48, 68, 74, 86, 93, 94, 95, 97, 104, 109	0 ~ 46	18 ~ 19	18
	Delinquent	6	39, 43, 67, 81, 82, 90	0 ~ 12	2 ~ 3	2
	Social Withdrawal	11	13, 42, 65, 69, 75, 80, 87, 88, 102, 103, 111	0 ~ 22	8 ~ 9	8
	Sex Problems	6	52, 60, 63, 73, 93, 96	0 ~ 12	3 ~ 4	3
	Cruel	7	5, 15, 16, 20, 21, 37, 57	0 ~ 14	3 ~ 4	3
	Schizoid-Obsessive	11	9, 18, 40, 66, 67, 70, 76, 84, 85, 91, 100	0 ~ 22	3 ~ 4	3

### Data collection

This cross-sectional survey was conducted in Fujian, China. With the teacher’s consent, we entered the classroom to explain the study’s significance to the children. We informed them that we needed to collect data on their screen time over a week and a questionnaire about problem behaviours. We assured the children that the survey would not affect their grades or teachers’ evaluations of them, as the study ensured their privacy. We invited them to take the questionnaire home, which included the purpose, significance, and considerations of the study, and promised to guarantee its anonymity. After obtaining written consent from the parents, we asked them to complete the questionnaire and have their child bring it to school for the teacher. Teachers did not know what the scores on the questionnaires mean.

The surveyors were primary school class teachers selected for this study. Prior to commencing the study, the researchers conducted a standardised training session for these teachers. Subsequently, the teachers distributed paper-based questionnaires to the children before the start of the school day, instructing them to take the questionnaires home with their primary caregivers. Over a period of one week, the primary caregivers observed and documented the children’s daily screen time and use of electronic devices outside of school hours. Based on these observations, the caregivers completed the questionnaire. In this study, parents recorded their children’s screen time over one week, which may have resulted in information bias. Our researchers emphasised to homeroom teachers the importance of instructing parents to fill out the questionnaire truthfully and provide objective responses, stating that the data would be used solely for research purposes. After completing the questionnaires, the children were instructed to return them to school for submission. It should be noted that the CBCL is a parent-report measure completed by the primary caregiver, who is knowledgeable about the child’s behaviour. The conclusions were drawn from data collected from October to November 2022.

### Data analysis

Statistical analysis was conducted using SPSS version 26.0 (Armonk, NY, IBM Corp.) The analysis consisted of both descriptive and inferential statistics. (1) Descriptive Statistics: Quantitative data with a normal distribution were represented using the mean and standard deviation (®*x* ± *s*). For non-normally distributed data such as CBCL scores and screen time, the median (*M*) and interquartile range (*IQR*) were used, and the *IQR* was calculated as *Q*_3_–*Q*_1_. Count data, specifically sociodemographic variables, are presented as frequency (*n*) and constituent ratio (*%*). (2) Inferential Statistics: (a) Correlation analysis: Spearman’s correlation coefficient was employed to investigate the relationship between screen time and problem behaviours. (b) Propensity Score Matching (PSM): PSM was used to assess whether children were overexposed to daily video content. A 1:1 matching ratio was utilised with the nearest neighbour method, a calliper value of 0.02, and a random number seed set of 1234. The Mann-Whitney U test was used to compare CBCL scores between the overexposed and non-overexposed groups before and after PSM. PSM is designed to reduce the effects of bias and confounding variables. (c) Univariate analysis: Differences in the total scores of problem behaviours among school-aged children were analysed based on individual characteristics. The Mann-Whitney *U* test was used for comparisons between two groups, while the Kruskal-Wallis *H* test was used for comparisons involving three or more groups. Additionally, the chi-square and Fisher’s exact tests were performed where appropriate. (d) Multivariate analysis: Considering fixed confounding variables, a generalised linear model (GLM) was used to examine the effects of daily video exposure time and excessive daily video exposure on problem behaviours. A logistic regression analysis was also conducted. Statistical significance was set at *p* < 0.05.

## Results

### Demographics

The sociodemographic characteristics and differences in the CBCL scores based on individual characteristics are presented in Table 2, while the utilisation of electronic products is presented in Table 3.

**Table 2:** Sociodemographic characteristics and differences in CBCL scores among individual characteristics (*n*=891)

Variable	Categories	*n *(*%*)	CBCL scores*/M*（*IQR*）	*Z*/*χ*^2^	*P*
Age, year	7	258(28.96)	11.00（19.00）	2.427	0.297
	8	471(52.86)	10.00（20.00）		
	9	162(18.18)	8.00（15.00）		
Sex	Male	468(52.53)	9.00（19.00）	0.742	0.458
	Female	423(47.47)	9.00（19.00）		
Grade	First grade	450(50.51)	10.00（21.00）	0.965	0.335
	Second grade	441(49.49)	9.00（17.00）		
Nation	Han nationality	884(99.21)	10.00（19.00）	1.075	0.282
	Other nationalities	7(0.79)	14.00（33.00）		
Only child	Yes	66(7.41)	10.00（18.75）	0.162	0.872
	No	825(92.59)	10.00（19.00）		
Overweight or obese	No	821(92.14)	10.00（19.00）	0.345	0.730
	Yes	70(7.86)	9.00（18.25）		
Sibling order	First	285(31.99)	13.00（19.00）	12.835	0.005
	Second	449(50.39)	9.00（19.00）		
	Third	142(15.94)	7.00（17.25）		
	Fourth and above	15(1.68)	9.00（17.00）		
Main caregiver	Parents	721(80.92)	9.00（19.00）	9.839	0.007
	Grandparents	120(13.47)	14.00（20.75）		
	Other	50(5.61)	5.00（22.50）		
Father 's educational level	Junior high school and below	536(60.16)	10.00（19.00）	0.078	0.962
	High school or technical secondary school	236(26.49)	9.00（20.00）		
	Junior college or above	119(13.36)	9.00（20.00）		
Father's occupation	Civil servants or employees of enterprises or institutions	63(7.07)	11.00（20.00）	8.200	0.085
	Worker	94(10.55)	10.50（22.50）		
	Peasant	131(14.70)	7.00（17.00）		
	Freelance work	357(40.07)	10.00（19.00）		
	Other occupations	246(27.61)	10.00（19.25）		
Mother 's educational level	Junior high school and below	562(63.08)	9.00（18.00）	3.171	0.205
	High school or technical secondary school	211(23.68)	12.00（22.00）		
	Junior college or above	118(13.24)	9.00（18.50）		
Mother's occupation	Civil servants or employees of enterprises or institutions	82(9.20)	11.00（20.00）	12.248	0.016
	Worker	43(4.83)	15.00（24.00）		
	Peasant	154(17.28)	8.00（17.25）		
	Freelance work	260(29.18)	10.00（18.00）		
	Other occupations	352(39.51)	9.00（20.00）		
Per capita monthly household income, RMB	<3000	233(26.15)	10.00（19.00）	1.528	0.676
	3000~4999	335(37.60)	10.00（18.00）		
	5000~7999	221(24.80)	8.00（18.50）		
	≥8000	102(11.45)	9.50（19.50）		

**Table 3:** Smartphone use among school-age children

Usage	Never (%)	Occasionally (%)	Sometimes (%)	Often (%)	Always (%)	Average scores
Learning	110(12.35)	319(35.80)	295(33.11)	128(14.37)	39(4.38)	2.63 ± 1.02
Socializing (connecting with others)	540(60.61)	205(23.01)	116(13.02)	25(2.81)	5(0.56)	1.60 ± 0.86
Browsing web pages	677(75.98)	148(16.61)	51(5.72)	12(1.35)	3(0.34)	1.33 ± 0.68
Playing mobile games	579(64.98)	218(24.47)	63(7.07)	22(2.47)	9(1.01)	1.50 ± 0.81
Watching movies and videos	206(23.12)	379(42.54)	209(23.46)	88(9.88)	9(1.01)	2.23 ± 0.95
Listening to music	210(23.57)	313(35.13)	237(26.60)	113(12.68)	18(2.02)	2.34 ± 1.04
Reading e-books	690(77.44)	131(14.70)	52(5.84)	13(1.46)	5(0.56)	1.33 ± 0.70
Taking pictures or shooting	386(43.32)	327(36.70)	144(16.16)	28(3.14)	6(0.67)	1.81 ± 0.86

### Screen time

**Fig. 1:**
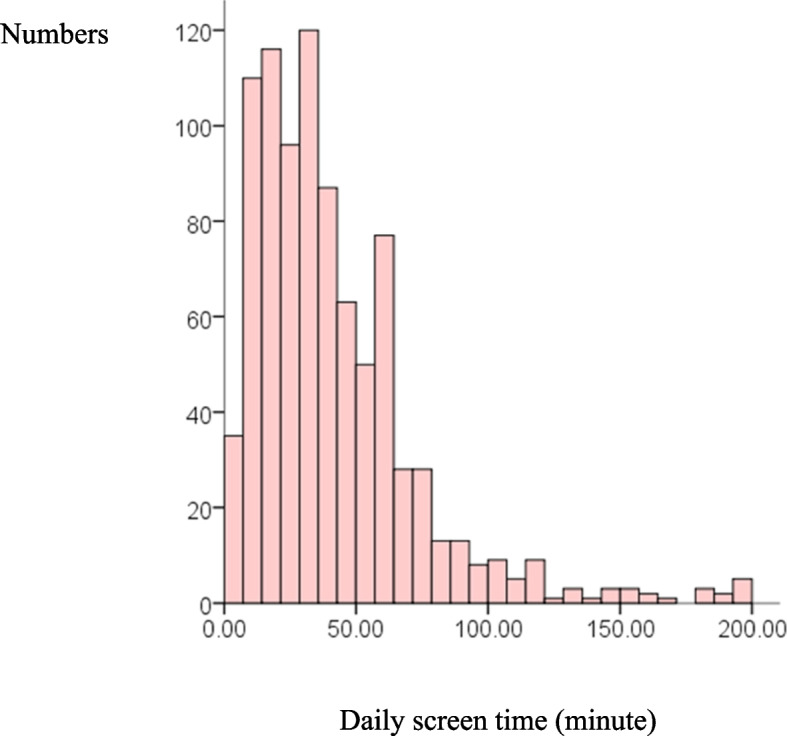
Frequency distribution histogram of school-age children’s daily screen time

The frequency distribution of school-age children’s daily screen time is presented in Fig. 1, which shows a skewed distribution. The median screen time for school-aged children was 34.29 (17.14, 55.71) min/day. Screen time exceeding 1 h per day was considered excessive [32, 39, 40]. Further, 17.06% (152/891) of the children were exposed to excessive screen time videos daily, including 9.43% (84/891) on weekdays and 34.90% (311/891) on weekends.

### Problem behaviours in school-age children

**Fig. 2:**
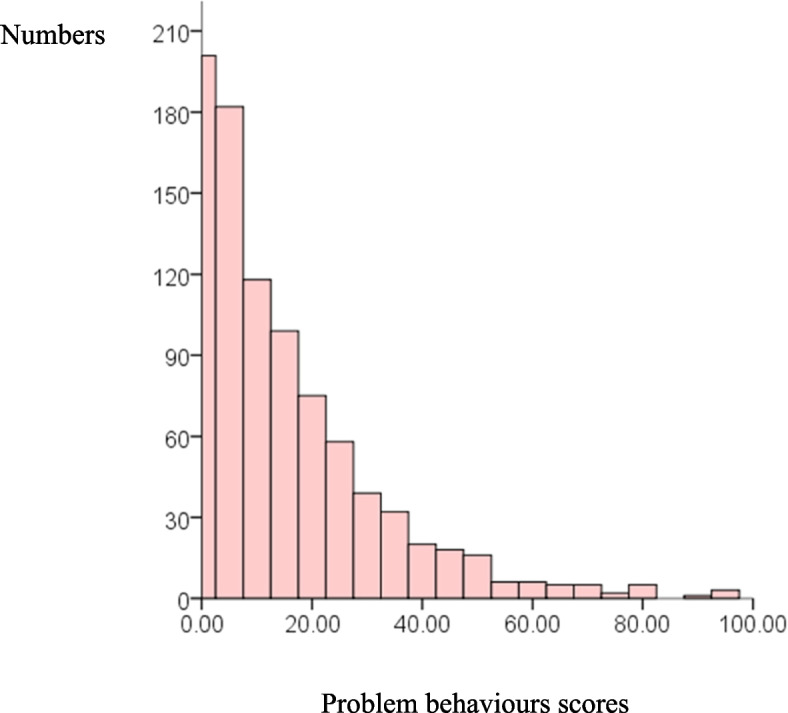
Frequency distribution histogram of school-age children’s problem behaviours scores

The frequency distribution of problem behaviours’ scores is shown in Fig. 2, which also indicates a skewed distribution. The total CBCL score of school-aged children was 10.00 (3.00, 22.00), and 10.00% had problem behaviours. The total CBCL score of boys was 10.00 (3.00, 22.00), and 10.68% exhibited problem behaviours. For girls, the total CBCL score was 9.00 (3.00, 22.00), and 9.22% exhibited problem behaviours. Notably, no significant differences were observed between boys and girls (*p* > 0.05). The total and dimensional scores are listed in Table 4.

**Table 4:** Total and dimensions scores of problem behaviours among school-age children and single-factor analysis of screen exposure on each dimension of problem behaviour (*n* = 891)

Gender	Item	M(IQR)	*n* (%)	Daily excessive screen exposure *n* (%)		χ ^2^	*p* value
				Yes	No		
Children	CBCL scores	10.00(19.00)	89(10.00)	-	-	-	-
Boys	CBCL scores	10.00(19.00)	50(10.68)	-	-	-	-
	Depressed	0.00(2.00)	18(3.85)	7(8.24)	11(2.87)	4.057	0.044
	Somatic Complaints	0.00(1.00)	3(0.64)	2(2.35)	1(0.26)	-	0.086
	Hyperactive	3.00(4.00)	25(5.34)	6(7.06)	19(4.96)	0.262	0.609
	Aggressive	3.00(6.00)	15(3.21)	6(7.06)	9(2.35)	3.570	0.059
	Delinquent	1.00(2.00)	16(3.42)	9(10.59)	7(1.83)	13.624	< 0.001
	Social Withdrawal	0.00(1.00)	26(5.56)	8(9.41)	18(4.70)	2.114	0.146
	Obsessive-compulsive	0.00(2.00)	26(5.56)	7(8.24)	19(4.96)	0.866	0.352
	Uncommunicative	0.00(1.00)	34(7.26)	6(7.06)	28(7.31)	0.007	0.935
	Schizoid	1.00(2.00)	32(6.84)	11(12.94)	21(5.48)	6.074	0.014
Girls	CBCL scores	9.00(19.00)	39(9.22)	-	-	-	-
	Depressed	2.00(3.00)	6(1.42)	2(2.99)	4(1.12)	-	0.243
	Somatic Complaints	0.00(2.00)	9(2.13)	4(5.97)	5(1.40)	3.665	0.056
	Hyperactive	2.00(5.00)	22(5.20)	10(14.93)	12(3.37)	13.015	< 0.001
	Aggressive	2.00(5.00)	11(2.60)	4(5.97)	7(1.97)	2.163	0.141
	Delinquent	0.00(0.00)	35(8.27)	8(11.94)	27(7.58)	1.41	0.235
	Social Withdrawal	1.00(2.00)	11(2.60)	5(7.46)	6(1.69)	5.325	0.021
	Sex Problems	0.00(1.00)	20(4.73)	3(4.48)	17(4.78)	0.000	> 0.999
	Cruel	0.00(0.00)	21(4.96)	7(10.45)	14(3.93)	3.786	0.052
	Schizoid-Obsessive	0.00(1.00)	26(6.15)	7(10.45)	19(5.34)	1.744	0.187

Note. There were 468 boys, of whom 85 were over-exposed and 383 were not. There were 423 girls, of whom 67 were over-exposed and 356 were not

### Relationship between excessive screen time and problem behaviours

Daily screen time (*r* = 0.228, *p* < 0.001), weekday screen time (*r* = 0.170, *p* < 0.001), and weekend screen time (*r* = 0.238, *p* < 0.001) were positively associated with the total score for problem behaviours. Before PSM, the total CBCL score of the overexposed group was significantly higher than that of the non-overexposed group (*Z* = 6.348, *p* < 0.001). A 1:1 PSM was used to control for baseline confounding factors in school-aged children, using all individual characteristics as control variables. After PSM, 290 school-aged children were included, with 145 in the daily overexposed group and 145 in the non-overexposed group. After PSM, the total CBCL score of the overexposed group remained significantly higher than that of the non-overexposed group (*Z* = 5.466, *p* < 0.001) (Table 5).

**Table 5:** Propensity score matching (PSM) analysis results

	Variable	Categories	*n*	CBCL scores/M(IQR)	Z	*P*
Before PSM	Daily excessive screen time	No	739	8.00(18.00)	6.348	< 0.001
		Yes	152	18.00(24.75)		
After PSM	Daily excessive screen time	No	145	8.00(16.00)	5.466	< 0.001
		Yes	145	18.00(24.00)		

A generalised linear model (GLM) was used to account for four variables (sibling order, father’s and mother’s occupation, and primary caregiver) with *p* < 0.100 in the univariate analysis as confounding variables in the model. The total CBCL score was taken as the dependent variable. We used the daily video exposure time (Model 1) and excessive daily video exposure (Model 2) as independent variables (Table 6). The likelihood ratio of the Omnibus test for Model 1 was *χ* ^*2*^ = 50.919, *p* < 0.001. The likelihood ratio of the Omnibus test for Model 2 was *χ* ^*2*^ = 70.783, *p* = 0.006. Confounding variables for Models 1 and 2, including sibling order (third vs. first and second vs. first) and mother’s occupation (worker vs. civil servant or employee of enterprises and institutions), were entered into the model. In other words, the risk of problem behaviours in the third (Model 1: *χ* ^*2*^ = 5.550; Model 2: *χ* ^*2*^ = 5.349) or second (Model 1: *χ* ^*2*^ = 7.773; Model 2: *χ* ^*2*^ = 6.371) child was lower than that in the first one (*p* < 0.05). Children whose mothers are workers (Model 1: *χ* ^*2*^ = 6.654; Model 2: *χ* ^*2*^ = 6.849) are at higher risk of developing problem behaviours than children whose mothers are civil servants or employees of public institutions (*p* < 0.05). After controlling for confounding variables, daily screen time in Model 1 and excessive daily video exposure in Model 2 were significant factors that affected problem behaviours. The results of Models 1 and Model 2 were consistent and robust (Table 7).

**Table 6:** Generalized linear model assignment

Variable	Variable name	Assignment
Dependent variable	Total score of CBCL	Continuous variable value
Independent variable	Sibling order	（0,0,0）= First,（1,0,0）= Second,（0,1,0）=Third,（0,0,1）= Fourth and above
	Father 's educational level	（0,0,0,0）= Civil servants or employees of enterprises or institutions,（1,0,0,0）= Worker,（0,1,0,0）= Peasant,（0,0,1,0）= Freelance work,（0,0,0,1）= Other occupations
	Mother 's educational level	（0,0,0,0）= Civil servants or employees of enterprises or institutions,（1,0,0,0）= Worker,（0,1,0,0）= Peasant,（0,0,1,0）= Freelance work,（0,0,0,1）= Other occupations
	Main caregiver	（0,0）= Parents,（1,0）= Grandparents,（0,1）= Other
	Daily screen exposure time	Raw value
	Daily excessive screen exposure	1= >1h/d, 0=≤1 h/d

**Table 7:** Generalized linear model analysis (*n*=891)

Model	Variable	Categories	*B-value*	*S.E.-value*	*95%Wald *	*Waldχ*^2^	*p value*
Model 1	Intercept		17.400	2.821	11.872~22.929	38.052	<0.001
	Daily screen exposure time		0.064	0.014	0.037~0.091	21.847	<0.001
	Sibling order	Fourth and above	−7.360	4.835	−16.836~2.117	2.317	0.128
		Third	−4.565	1.938	−8.363~−0.767	5.550	0.018
		Second	−3.891	1.396	−6.626~−1.156	7.773	0.005
	Father's occupation	Other occupations	−0.775	2.712	−6.089~4.540	0.082	0.775
		Freelance work	−1.687	2.571	−6.725~3.351	0.431	0.512
		Peasant	−4.516	3.767	−11.899~2.867	1.437	0.231
		Worker	−3.228	3.162	−9.426~2.970	1.042	0.307
	Mother's occupation	Other occupations	−0.853	2.369	−5.496~3.791	0.129	0.719
		Freelance work	−1.400	2.404	−6.112~3.312	0.339	0.560
		Peasant	0.190	3.432	−6.536~6.916	0.003	0.956
		Worker	9.631	3.734	2.313~16.949	6.654	0.010
	Main caregiver	Other	0.912	2.643	−4.268~6.091	0.119	0.730
		Grandparents	1.730	1.816	−1.829~5.289	0.908	0.341
Model 2	Intercept		17.727	2.764	12.309~23.145	41.122	<0.001
	Daily excessive screen exposure	Yes(>1h/d)	10.400	1.597	7.271~13.529	42.426	<0.001
	Sibling order	Fourth and above	−7.468	4.778	−16.832~1.897	2.443	0.118
		Third	−4.433	1.917	−8.189~−0.676	5.349	0.021
		Second	−3.484	1.381	−6.190~−0.779	6.371	0.012
	Father's occupation	Other occupations	−0.796	2.681	−6.051~4.459	0.088	0.767
		Freelance work	−1.242	2.543	−6.227~3.742	0.239	0.625
		Peasant	−4.515	3.725	−11.816~2.786	1.469	0.225
		Worker	−3.014	3.127	−9.143~3.116	0.929	0.335
	Mother's occupation	Other occupations	-.549	2.343	−5.142~4.044	0.055	0.815
		Freelance work	−1.255	2.373	−5.906~3.396	0.280	0.597
		Peasant	0.513	3.394	−6.139~7.164	0.023	0.880
		Worker	9.661	3.691	2.426~16.895	6.849	0.009
	Main caregiver	Other	0.248	2.616	−4.879~5.375	0.009	0.924
		Grandparents	1.827	1.795	−1.692~5.345	1.036	0.309

Through sex stratification, we found statistically significant differences in depression, delinquency, and schizoid among boys (*p* < 0.05) and in hyperactivity and social withdrawal among girls (*p* < 0.05) (Table 4**)**. Based on this, the occurrence of problem behaviours in all dimensions of school-age children (yes assigned 1, no assigned 0) was used as the dependent variable, and excessive daily video exposure (more than 1 h per day assigned 1, otherwise 0) was used as the independent variable. We found that excessive video exposure promoted depression, aggression, delinquency, social withdrawal, and schizoid among boys (*p* < 0.05) and hyperactivity and social withdrawal among girls (*p* < 0.05) (Table 8).

**Table 8:** Logistic regression analysis of screen exposure on each dimension of problem behaviour

Gender	Independent variable	Dependent variable	*β-value*	*S.E.-value*	*Waldχ*^2^	*p value*	*OR*	*95％CI*
Boys	Daily excessive screen exposure	Depressed	1.753	0.661	7.035	0.008	5.770	1.580~21.069
		Hyperactive	0.439	0.566	0.602	0.438	1.551	0.512~4.704
		Aggressive	1.423	0.641	4.931	0.026	4.148	1.182~14.562
		Delinquent	2.686	0.723	13.812	<0.001	14.677	3.559~60.520
		Social Withdrawal	1.188	0.521	5.202	0.023	3.280	1.182~9.103
		Obsessive-compulsive	0.914	0.523	3.057	0.080	2.494	0.895~6.949
		Uncommunicative	0.141	0.530	0.071	0.791	1.151	0.407~3.256
		Schizoid	1.060	0.453	5.483	0.019	2.887	1.189~7.014
Girls	Daily excessive screen exposure	Depressed	0.888	1.351	0.432	0.511	2.429	0.172~34.297
		Somatic Complaints	1.347	0.801	2.832	0.092	3.848	0.801~18.483
		Hyperactive	1.633	0.544	9.009	0.003	5.117	1.762~14.862
		Aggressive	1.306	0.877	2.221	0.136	3.693	0.662~20.586
		Delinquent	0.380	0.471	0.651	0.420	1.462	0.581~3.681
		Social Withdrawal	1.873	0.795	5.550	0.018	6.506	1.370~30.901
		Sex Problems	−0.596	0.773	0.594	0.441	0.551	0.121~2.509
		Cruel	1.058	0.554	3.640	0.056	2.880	0.972~8.538
		Schizoid-Obsessive	0.965	0.539	3.202	0.074	2.624	0.912~7.547

Note. Due to the small sample size, no statistical analysis was made for the Somatic Complaints of boys

The datasets generated or analysed during the current study are not publicly available because this article is part of the author’s master’s thesis, which is subject to a confidentiality period of 2 years. The data associated with our study have not been deposited in a publicly accessible repository because they are confidential. Although these datasets are not currently suitable for publication, they are available from the corresponding author upon a reasonable request.

## Discussion

In the present study, 10.00% of the children exhibited problem behaviours. The incidence of problem behaviours among school-aged children is low. This finding closely aligns with a survey conducted among 2,386 children aged 6–11 years in Shandong Province, where the detection rate was 9.4% [4]. However, this is notably lower than the 23.7% rate reported in Guizhou Province [41]. Additionally, the result of our analysis did not reveal any statistically significant difference in problem behaviours between boys and girls (*p* > 0.05), contradicting the findings of Qi [42]. One possible explanation for this discrepancy could be the narrower age range (6 to 9 years) of children in our study compared to those in other studies. At this stage, children’s physical functions are still developing, and they are in the process of synchronising their self-awareness with their behaviours. A previous study [43] identified age differences between boys and girls, leading to distinct developmental stages and, consequently, unequal manifestations of problem behaviours between sexes. Notably, the predominant problem behaviours among boys in our study were depression, aggression, delinquency, social withdrawal, and schizoid tendencies. Among girls, hyperactivity and social withdrawal were the primary concerns. These findings are consistent with those of previous studies [44, 45]. In addition, our study highlighted the importance of considering sibling rank and maternal occupation when examining problem behaviours in children. Therefore, future research should explore these factors in greater depth to gain a more comprehensive understanding of the complex interplay between sibling rank, socioeconomic factors, and child development. Furthermore, it is essential to consider incorporating other key factors related to problem behaviours, such as personality traits. In summary, while our findings provide valuable insights into the prevalence and nature of problem behaviours among children, further research is warranted to elucidate the underlying mechanisms and identify effective intervention strategies.

Moreover, we identified sibling rank as a significant factor influencing children’s problem behaviours. Specifically, the presence of a second or third child within the family may have a notable impact on the behaviour of the firstborn. It is plausible that parents often devote more time and energy to caring for their younger children, which can lead the firstborn to exhibit psychological distress or problem behaviours as a means of seeking attention. Furthermore, our findings suggest that the mother’s occupation plays a crucial role in determining the risk of problem behaviours in children. Children whose mothers were engaged in manual labour or blue-collar jobs exhibited a higher prevalence of problem behaviours compared to those whose mothers were civil servants or employees of enterprises and institutions (*p* < 0.05). These results underscore the importance of considering the familial actual situation, particularly sibling relationships and parental occupations when assessing and addressing problem behaviours in children. It must be acknowledged that the cross-sectional design inherently limited our ability to explain causality. Future studies should consider employing designs that can confirm causality, elucidating the mechanisms underlying these associations and exploring potential intervention strategies tailored to these specific risk factors.

Furthermore, our findings revealed that 17.06% of the children engaged in excessive screen time, which is lower than the findings of a 2016–2017 study involving Chinese primary school students [46]. Additionally, daily, weekday, and weekend screen time were positively associated with problem behaviours. Primarily, smartphones were used as learning aids, with parents providing electronic devices to facilitate online lessons, search for answers, and enrich knowledge. This way can enhance learning motivation and enjoyment; however, parents must carefully monitor the duration and content of their children’s screen exposure. The foremost priority should be placed on limiting cumulative screen time, while selecting educational programs and avoiding non-child-directed content [47]. Specifically, a minority of children reported using smartphones for entertainment activities, such as listening to music (12.68%) and watching movies or television (9.88%). Given children’s immature physical and mental development and sensitivity to electronics, early and prolonged exposure to electronic devices may hinder their ability to relax physically and mentally during leisure time. This, in turn, increases the likelihood of Internet addiction. Internet addiction has a significant negative impact on child physical and mental health, reducing their sleep and exercise time [48]. Furthermore, our findings revealed that 9.43% and 34.90% of children exceeded the recommended screen exposure limits on weekdays and weekends, respectively. This difference is similar to the findings of a previous study conducted in Holland [49], suggesting that the prevalence of social media use was 37.7% on a weekday and 59.6% on a weekend day among adolescents because of low socioeconomic position. In conclusion, although electronic devices can serve as valuable learning tools, their misuse for entertainment purposes can have detrimental effects on children’s physical and mental health. Parents play a crucial role in monitoring and guiding children’s screen time habits to ensure a balanced approach that promotes healthy development. Indeed, this study’s scope was limited to children living in Fujian; future studies should include children from other regions to validate these findings.

Prolonged exposure to screens and excessive screen time were identified as significant contributors to problem behaviours among school-age children, consistent with previous research findings [24, 25, 50]. Promoting companionship from caregivers and emotional regulation skills may serve as protective factors for young children who are highly exposed to screen time and thus at risk of problem behaviours [12]. Therefore, we suggest that caregivers and other family members adopt more serious and cautious attitudes toward children’s screen exposure and implement appropriate digital-related parenting practices [51]. Because, in this digital age, children inevitably need to be exposed to electronic products. Parents need to not only see the benefits of electronic products for children’s life and learning, but also be alert to their negative impact on children. Moreover, excessive screen time directly reduces sleep duration and interferes with the release of melatonin, a hormone crucial for sleep onset. This can stimulate the excitability of the central nervous system, disrupt sleep rhythms, decrease deep sleep time, and ultimately reduce sleep quality [52, 53]. These disruptions in sleep patterns can contribute to problem behaviours in children. Our findings underscore the importance of limiting screen time, promoting healthy parent-child interactions, and ensuring adequate sleep in school-age children to mitigate the risk of problem behaviours. Future research should aim to develop interventions targeting these modifiable risk factors to promote positive behavioural outcomes in this population.

In practice terms, our study provides a reference for children’s screen-related health education: (a) encouraging parents to provide high-quality companionship to children. For example, parents should increase their engagement in parent-child activities, respond to their children’s needs, reduce screen time, and reduce the occurrence of problem behaviours. (b) Encouraging parents to adopt more serious and cautious attitudes towards their children’s use of electronic devices is important. For example, parents should recognise and respect that children growing up in the digital age are accustomed to electronic devices. At the same time, parents must clearly understand the pros and cons of using electronic devices. (c) Educators can use the CBCL to monitor and tailor interventions based on students’ screen time and its effects. (d) Schools could implement programs to educate about screen time risks and promote healthier digital habits.

## Conclusion

In a large sample of data from Fujian, we found that the average screen time for school-age children was 34.29 min per day (SD = 38.57). Additionally, 17.06% (152/891) of the children were exposed to excessive video content daily, with 9.43% (84/891) on weekdays and 34.90% (311/891) on weekends. Daily, weekday, and weekend screen time were all positively correlated with the total score for problem behaviours. Excessive video exposure was associated with exacerbated depression, aggression, delinquency, social withdrawal, and schizoid tendencies among boys, and hyperactivity and social withdrawal among girls. Therefore, we plan to develop a family play intervention to reduce the adverse effects of screen time on children’s problem behaviours in follow-up studies. Parents are encouraged to limit screen time and engage in interactive activities to mitigate behavioural risks. Educators can use the CBCL to monitor and tailor interventions based on students’ screen time and its effects. Schools could implement programs to educate about screen time risks and promote healthier digital habits.

### Limitations

The present study has several limitations that should be considered when interpreting the findings. First, the cross-sectional design prevents us from inferring causality among the variables accounting for potential unknown confounders. Second, while we endeavoured to include key factors influencing problem behaviours, other relevant factors, such as personality traits, were not considered. Additionally, the reliance on observational record methods to collect data from children over a one-week period may have introduced information bias owing to the discontinuous nature of the data collection timeframe. Finally, the scope of the study was limited to children in Fujian due to time, personnel, and material resource constraints, which limited the generalisability of our findings. Therefore, future research should aim to broaden the scope of the investigation by including diverse cities and conducting multi-centre large-sample surveys. Employing random stratified sampling techniques would allow for more comprehensive explorations and comparisons of problem behaviours among children. These approaches would enhance the generalisability of the findings and provide a more nuanced understanding of the factors contributing to problem behaviours in children.

## Supplementary Information

Supplementary Material 1.

Supplementary Material 2.

## Data Availability

The datasets generated and/or analyzed during the current study are not publicly available due to the fact that this article is part of the author’s master’s thesis, which is subject to a confidentiality period of 2 years. The data associated with our study has not been deposited in a publicly accessible repository because the data used is confidential. While the datasets are not currently suitable for publication, they are available from the corresponding author upon a reasonable request.
