# Supplementary material for: Excessive screen time and problem behaviours among school-age children in Fujian, China: a cross-sectional study
Source: BMC Public Health. 2025 Feb 18;25:666. doi: 10.1186/s12889-025-21795-4 (PMC11837368; doi:10.1186/s12889-025-21795-4)
Supplement: Supplementary file 1 — Supplementary Material 1. [file 12889_2025_21795_MOESM1_ESM.docx]

**Excessive screen time and problem behaviours among school-age children in Fujian, China: a cross-sectional study**

**Smartphone Use Questionnaire (SUQ)**

What is your child's main use of electronic products?

Please tick "√" in the appropriate place.

| Usage | Never | Occasionally | Sometimes | Often | Always |
| --- | --- | --- | --- | --- | --- |
| Learning |  |  |  |  |  |
| Socializing (connecting with others) |  |  |  |  |  |
| Browsing web pages |  |  |  |  |  |
| Playing mobile games |  |  |  |  |  |
| Watching movies and videos |  |  |  |  |  |
| Listening to music |  |  |  |  |  |
| Reading e-books |  |  |  |  |  |
| Taking pictures or shooting |  |  |  |  |  |

**Screen Time Questionnaire (STQ)**

For one week, how many minutes per day did your child spend on the following activities?

After observing the child's behavior every day, you can **record the minutes** in the space timely and truthfully. Thank you.

| Projects | Monday | Tuesday | Wednesday | Thursday | Friday | Saturday | Sunday |
| --- | --- | --- | --- | --- | --- | --- | --- |
| Watching TV |  |  |  |  |  |  |  |
| Using electronic devices such as computers or mobile phones |  |  |  |  |  |  |  |

**The Child Behavior Checklist (CBCL)**

Please rate your child's behavior during the past six months.

The parents score the items on three-step response scales, with 0 indicating that the item is not true of the child, 1 indicating that the item is somewhat or sometimes true of the child, and 2 indicating that it is very true or often true of the child.

| Items | 0 | 1 | 2 |
| --- | --- | --- | --- |
| 1. Acts too young |  |  |  |
| 2. Allergy |  |  |  |
| 3. Argues |  |  |  |
| 4. Asthma |  |  |  |
| 5. Acts like opposite sex |  |  |  |
| 6. Encopresis |  |  |  |
| 7. Brags |  |  |  |
| 8. Can't concentrate |  |  |  |
| 9. Obsessions |  |  |  |
| 10. Hyperactive |  |  |  |
| 11. Clings to adults |  |  |  |
| 12. Lonely |  |  |  |
| 13. Confused |  |  |  |
| 14. Cries much |  |  |  |
| 15. Cruel to animals |  |  |  |
| 16. Cruel to others |  |  |  |
| 17. Daydreams |  |  |  |
| 18. Harms self |  |  |  |
| 19. Demands attention |  |  |  |
| 20. Destroys own things |  |  |  |
| 21. Destroys things belonging to others |  |  |  |
| 22. Disobedient at home |  |  |  |
| 23. Disobedient at school |  |  |  |
| 24. Doesn't eat well |  |  |  |
| 25. Poor peer relations |  |  |  |
| 26. Lacks guilt |  |  |  |
| 27. Jealous |  |  |  |
| 28. Eats nonfood |  |  |  |
| 29. Fears |  |  |  |
| 30. Fears school |  |  |  |
| 31. Fears own impulses |  |  |  |
| 32. Needs to be perfect |  |  |  |
| 33. Feels unloved |  |  |  |
| 34. Feels persecuted |  |  |  |
| 35. Feels worthless |  |  |  |
| 36. Accident prone |  |  |  |
| 37. Fighting |  |  |  |
| 38. Is teased |  |  |  |
| 39. Bad friends |  |  |  |
| 40. Auditory hallucination |  |  |  |
| 41. Impulsive |  |  |  |
| 42. Likes to be alone |  |  |  |
| 43. Lies, cheats |  |  |  |
| 44. Bites nails |  |  |  |
| 45. Nervous |  |  |  |
| 46. Twitches |  |  |  |
| 47. Nightmares |  |  |  |
| 48. Unliked |  |  |  |
| 49. Constipated |  |  |  |
| 50. Anxious |  |  |  |
| 51. Dizziness |  |  |  |
| 52. Feels guilty |  |  |  |
| 53. Overeats |  |  |  |
| 54. Overtired |  |  |  |
| 55. Overweight |  |  |  |
| 56a. Pains |  |  |  |
| 56b. Headaches |  |  |  |
| 56c. Nausea |  |  |  |
| 56d. Eye problems |  |  |  |
| 56e. Rashes |  |  |  |
| 56f. Stomach problems |  |  |  |
| 56g. Vomits |  |  |  |
| 56h. Other diseases |  |  |  |
| 57. Attacks people |  |  |  |
| 58. Picking |  |  |  |
| 59. Public masturbation |  |  |  |
| 60. Excess masturbation |  |  |  |
| 61. Poor school work |  |  |  |
| 62. Clumsy |  |  |  |
| 63. Prefers older children |  |  |  |
| 64. Prefers younger children |  |  |  |
| 65. Won't talk |  |  |  |
| 66. Compulsions |  |  |  |
| 67. Runs away |  |  |  |
| 68. Screams |  |  |  |
| 69. Secretive |  |  |  |
| 70. Visual hallucination |  |  |  |
| 71. Self-conscious |  |  |  |
| 72. Sets fires |  |  |  |
| 73. Sex problems |  |  |  |
| 74. Shows off |  |  |  |
| 75. Shy, timid |  |  |  |
| 76. Sleeps little |  |  |  |
| 77. Sleeps much |  |  |  |
| 78. Smears feces |  |  |  |
| 79. Speech problem |  |  |  |
| 80. Stares blankly |  |  |  |
| 81. Steals at home |  |  |  |
| 82. Steals outside home |  |  |  |
| 83. Hoarding |  |  |  |
| 84. Strange behavior |  |  |  |
| 85. Strange ideas |  |  |  |
| 86. Stubborn |  |  |  |
| 87. Moody |  |  |  |
| 88. Sulks |  |  |  |
| 89. Suspicious |  |  |  |
| 90. Swears |  |  |  |
| 91. Suicidal talk |  |  |  |
| 92. Walks, talks in sleep |  |  |  |
| 93. Excess talk |  |  |  |
| 94. Teases |  |  |  |
| 95. Temper tantrums |  |  |  |
| 96. Sex preoccupation |  |  |  |
| 97. Threatens people |  |  |  |
| 98. Thumb sucking |  |  |  |
| 99. Too neat |  |  |  |
| 100. Can't sleep |  |  |  |
| 101. Truant |  |  |  |
| 102. Slow moving |  |  |  |
| 103. Sad |  |  |  |
| 104. Loud |  |  |  |
| 105. Alcohol, drugs |  |  |  |
| 106. Vandalism |  |  |  |
| 107. Wets self |  |  |  |
| 108. Wets bed |  |  |  |
| 109. Whining |  |  |  |
| 110. Wishes to be opposite sex |  |  |  |
| 111. Withdrawn |  |  |  |
| 112. Worrying |  |  |  |
| 113. Your child also has other problems not mentioned above. |  |  |  |
