# Supplementary material for: Excessive screen time and problem behaviours among school-age children in Fujian, China: a cross-sectional study
Source: BMC Public Health. 2025 Feb 18;25:666. doi: 10.1186/s12889-025-21795-4 (PMC11837368; doi:10.1186/s12889-025-21795-4)
Supplement: Supplementary file 2 — Supplementary Material 2. [file 12889_2025_21795_MOESM2_ESM.docx]

**STROBE flow diagram**
